# Transcriptome response to pollutants and insecticides in the dengue vector *Aedes aegypti *using next-generation sequencing technology

**DOI:** 10.1186/1471-2164-11-216

**Published:** 2010-03-31

**Authors:** Jean-Philippe David, Eric Coissac, Christelle Melodelima, Rodolphe Poupardin, Muhammad Asam Riaz, Alexia Chandor-Proust, Stéphane Reynaud

**Affiliations:** 1 Laboratoire d'Ecologie Alpine (LECA, UMR 5553 CNRS - Université Grenoble), France

## Abstract

**Background:**

The control of mosquitoes transmitting infectious diseases relies mainly on the use of chemical insecticides. However, mosquito control programs are now threatened by the emergence of insecticide resistance. Hitherto, most research efforts have been focused on elucidating the molecular basis of inherited resistance. Less attention has been paid to the short-term response of mosquitoes to insecticides and pollutants which could have a significant impact on insecticide efficacy. Here, a combination of LongSAGE and Solexa sequencing was used to perform a deep transcriptome analysis of larvae of the dengue vector *Aedes aegypti *exposed for 48 h to sub-lethal doses of three chemical insecticides and three anthropogenic pollutants.

**Results:**

Thirty millions 20 bp cDNA tags were sequenced, mapped to the mosquito genome and clustered, representing 6850 known genes and 4868 additional clusters not located within predicted genes. Mosquitoes exposed to insecticides or anthropogenic pollutants showed considerable modifications of their transcriptome. Genes encoding cuticular proteins, transporters, and enzymes involved in the mitochondrial respiratory chain and detoxification processes were particularly affected. Genes and molecular mechanisms potentially involved in xenobiotic response and insecticide tolerance were identified.

**Conclusions:**

The method used in the present study appears as a powerful approach for investigating fine transcriptome variations in genome-sequenced organisms and can provide useful informations for the detection of novel transcripts. At the biological level, despite low concentrations and no apparent phenotypic effects, the significant impact of these xenobiotics on mosquito transcriptomes raise important questions about the 'hidden impact' of anthropogenic pollutants on ecosystems and consequences on vector control.

## Background

During the past 60 years, the amount of anthropogenic xenobiotics released into natural ecosystems has dramatically increased. Although the effect of these chemicals on human health is intensively studied, their impact on other organisms remains poorly understood. Because pollutants often accumulate in fresh-water bodies and sediments [1], their impact on wetland fauna is of importance for these ecosystems. Among aquatic arthropods found in wetlands, mosquitoes are distributed worldwide and are often exposed to anthropogenic pollutants and insecticides during their aquatic larval stage. Indeed insecticides are often deliberately introduced into the mosquito habitat in the fight against the many human diseases they transmit (e.g. malaria, dengue fever, yellow fever and filariasis) [2]. As a consequence mosquito control programs are now threatened by the selection of mosquito populations resistant to these chemical insecticides [3]. Differential gene transcription in insecticide-resistant mosquitoes has been frequently used to identify genes putatively involved in inherited metabolic resistance mechanisms [4-7]. For that purpose most approaches used cDNA microarrays and were often focused on genes encoding enzymes potentially involved in the bio-transformation of insecticides molecules [8,9], although recent findings suggest that the differential expression of other transcripts may also contribute to insecticide tolerance [4,10]. Less attention has been paid to the short term transcriptome response of insects to xenobiotics, though this may lead to the discovery of novel molecular mechanisms contributing to insecticide tolerance [11-13]. We recently demonstrated that exposing mosquito larvae to low concentrations of pollutants for a few hours can increase their tolerance to chemical insecticides, possibly due to an alteration of the expression of detoxification enzymes [11,12]. In this context, understanding cross responses of mosquitoes to insecticides and pollutants at the whole transcriptome level may ultimately lead to improvements in vector control strategies by optimizing insecticide treatments in polluted areas [7]. Moreover, deciphering transcriptome response of mosquitoes to anthropogenic xenobiotics may identify genes involved in chemical stress response that were not detected by standard toxicological studies.

Today, quantitative transcriptomic methods are diversified and divided into two kind of technology: 'closed' and 'open' techniques depending on genome annotation constraints [14,15]. In 'closed' technologies, gene expression microarrays are the standard method used for transcriptome analysis. However, this type of technology does not allow the characterization and analysis of new transcripts and suffers from various technical biases such as non-specific hybridization and insufficient signal for low expressed genes. In contrast, 'open' transcriptome analyses based on the sequencing of either ESTs or short cDNA tags, like Serial Analysis of Gene Expression (SAGE) [16], LongSAGE [17] and Massive Parallel Signature Sequencing (MPSS) [18] can measure the transcript level of both known and unknown genes [19]. The short cDNA tags obtained by LongSAGE or MPSS can directly be mapped to the genome sequence, allowing the identification of new transcripts [15]. Because these sequencing techniques do not target a defined portion of cDNAs, these approaches are not optimized for the deep analysis of transcriptome variations [20]. Recently, a combination of LongSAGE and Solexa sequencing technology, leading to the production and sequencing of millions of tags on a defined region of cDNAs, has been used to characterize mouse hypothalamus transcriptome [15]. To our knowledge, this new method, called Digital Gene Expression Tag Profiling (DGETP) has never been used to compare whole transcriptome variations of a non-mammalian organism in different environmental conditions.

Here, we used the DGETP approach to perform a deep transcriptome analysis of larvae of the mosquito *Aedes aegypti *exposed to different anthropogenic xenobiotics. We examined the effect of sublethal doses of three pollutants likely to be found in wetlands (the herbicide atrazine, the polycyclic aromatic hydrocarbon fluoranthene and the heavy metal copper) and three chemical insecticides used for mosquito control (the pyrethroid permethrin, the neonicotinoid imidacloprid and the carbamate propoxur). This approach was suitable for investigating deep transcriptome variations in mosquitoes and identified several loci with high transcription signal not previously identified in mosquito genome. At the biological level, the transcript levels of many genes were affected by xenobiotic exposure. Several genes and protein families responding to individual or multiple xenobiotics were identified, unraveling the complexity of xenobiotic-response in mosquitoes and identifying genes potentially involved in insecticide tolerance or biological interactions between insecticides and pollutants.

## Results

### Sequencing, mapping and clustering of cDNA tags

By sequencing 7 cDNA tag libraries from mosquito larvae exposed to different xenobiotics, a total of 29.45 million reads (100% of total reads) corresponding to 726,269 distinct 20-mer tags were obtained (Table 1). By removing any tag represented by less than 20 reads across all libraries, background filtering slightly reduced the total number of reads to 28.12 million (95.5%) but greatly reduced the number of distinct tags to 33,037. Among them, 15,253 distinct tags were successfully mapped onto the *Ae. aegypti *genome at a unique genomic location without mismatch, representing 15.2 million reads (51.6%). Among successfully mapped tags, 9,812 distinct tags (12.59 million reads, 42.7%) were mapped to 6,850 predicted genes while the remaining reads (8.9%) were mapped outside gene boundaries (see methods).

**Table 1:** Sequencing statistics

**Reads**		**Ctrl (×10^6^)**	**Copper (×10^6^)**	**Fluo (×10^6^)**	**Atraz (×10^6^)**	**Propo (×10^6^)**	**Perm (×10^6^)**	**Imida (×10^6^)**	**Mean (×10^6^)**	**Total (×10^6^)**	**% Total**	**Distinct tags**
			
Sequenced		4.35	4.30	4.41	2.75	3.88	4.90	4.85	4.21	29.45	100	726 269
Filtered from background		4.16	4.10	4.21	2.63	3.72	4.68	4.62	4.02	28.12	95.5	33 037
Mapped to genome		2.27	2.31	2.29	1.42	1.80	2.63	2.48	2.17	15.20	51.6	15 253
Mapped to genes		1.89	1.93	1.87	1.19	1.49	2.19	2.03	1.80	12.59	42.7	9 812

Reads filtered from background represent tags showing > 20 reads across all conditions. Reads mapped to genome represent tags mapped to a unique genomic location without mismatch. Reads mapped to genes represent tags filtered from background and mapped to predicted genes. Ctrl: controls; Copper: exposed to copper sulfate; Fluo: exposed to fluoranthene; Atraz: exposed to atrazine; Propo: exposed to propoxur; Perm: exposed to permethrin; Imida: exposed to imidacloprid.

Clustering analysis of 20-mer cDNA tags successfully mapped to mosquito genome allowed us to identify a total of 13,118 distinct clusters including 8,250 clusters associated to predicted genes. Distribution of the total number of reads across genes, clusters and tags (Additional file 1: Suppl. Figure 1) spanned more than 4 orders of magnitude with most genes/clusters being represented by 25 to 5000 reads. Median total number of reads per gene, cluster, tag and cluster not mapped within predicted gene were 217, 124, 101 and 79 respectively.

### Quantitative transcription data obtained from cDNA tags

**Figure 1:**
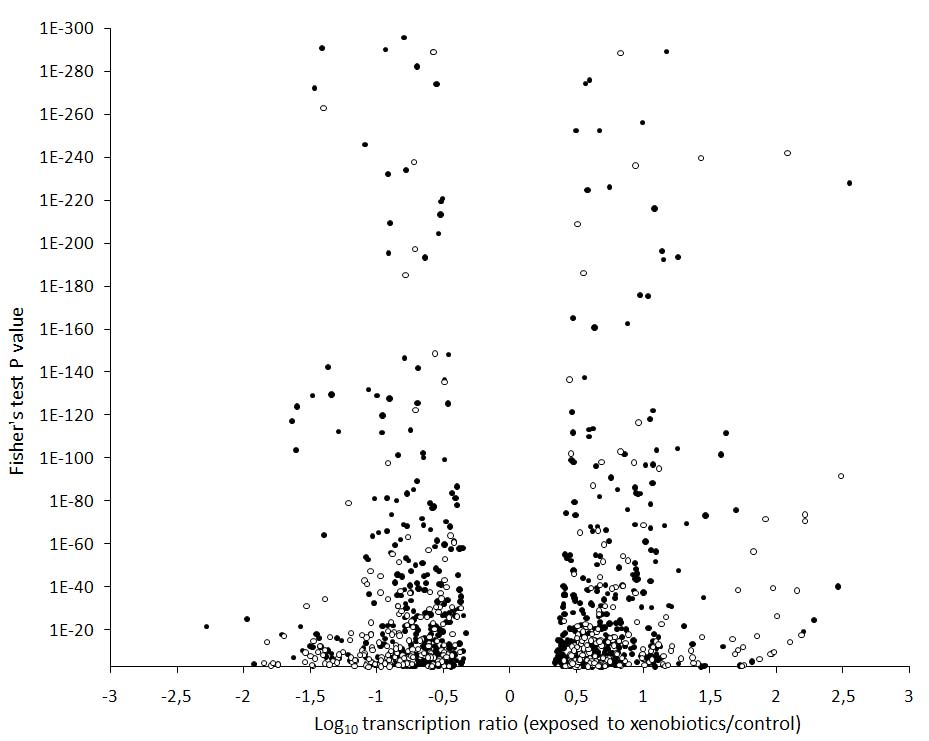
**Distribution and significance of transcription variations in mosquito larvae exposed to xenobiotics**. Transcription ratios of genes are shown as black dots while genomic clusters not mapped within genes are shown as white dots. Differential transcription is indicated as a function of both log_10 _transcription ratios (exposed to xenobiotics/controls) and Fisher's test P_values_. Only the transcription ratios of 453 genes and 250 clusters showing a Fisher's test P_value _< 0.001 in at least one condition are shown.

Analysis of transcription levels in mosquito larvae exposed to each xenobiotic was performed at the gene level for tags mapped within predicted genes (i.e. gathering all tags mapped within each gene) and at the cluster level for tags not mapped within predicted genes (i.e. gathering all tags mapped within each cluster). This analysis identified 453 genes and 225 additional clusters with a mean transcript ratio (TR) significantly > 2-fold in either direction in at least 1 condition (Fisher's test P_value _< 10^-3 ^after multiple testing correction). Overall distribution of TRs and their associated P_values _revealed a well-balanced distribution between over- and under transcription with TRs ranging from 600-fold under transcription to more than 2000-fold over transcription compared with controls (Figure 1 and Additional file 2: Suppl. Table 1). Cross-validation of TRs with real-time quantitative RT-PCR on 14 genes (Additional file 3: Suppl. Figure 2) revealed a good correlation of TRs obtained from the two techniques (r = 0.71 and P = 4.16 E-05), although the DGETP method often produced higher TRs (in either direction) than real-time quantitative RT-PCR.

### Overall transcriptome variations across treatments

Global analysis of transcriptome variations between mosquito larvae exposed to each xenobiotic revealed that the proportion of genes/clusters differently transcribed varied greatly between treatments (Table 2). This proportion ranged from 0.26% to 3.94% of all detected genes/clusters for permethrin and propoxur respectively. No correlation was found between the number of genes/clusters differentially transcribed in each treatment and the number of reads sequenced or the number of cDNA tags successfully mapped to genome, suggesting an accurate normalization across all libraries. When considering organic xenobiotics (all but copper), the number of genes/clusters differentially transcribed for each treatment was significantly positively correlated with the molarity of the xenobiotic used for larval exposure, (r = 0.89 and P < 0.05). This overall positive correlation revealed that despite the different nature of xenobiotics, increasing the number of organic molecules lead to an increase in the number of genes/cluster differentially transcribed. Principal component analysis (PCA) based on TRs of genes/clusters differentially transcribed revealed similar transcriptome variations of mosquito larvae exposed to the two chemical insecticides propoxur and imidacloprid and the polycyclic aromatic hydrocarbon fluoranthene (Additional file 4: Suppl. Figure 3). Conversely, transcriptome variations of larvae exposed to the insecticide permethrin, the herbicide atrazine and copper were more specific.

**Table 2:** Genes and clusters differentially transcribed after xenobiotic exposure

**Genes/clusters differentially transcribed**		**Copper**		**Fluo**		**Atraz**		**Propo**		**Perm**		**Imida**	
		**N**	**%**	**N**	**%**	**N**	**%**	**N**	**%**	**N**	**%**	**N**	**%**
						
Total genes and additional clusters		71	0.61	141	1.20	98	0.84	462	3.94	31	0.26	361	3.08
Total genes		49	0.72	86	1.26	60	0.88	318	4.64	20	0.29	239	3.49
Over-transcribed		46	0.67	50	0.73	25	0.36	130	1.90	16	0.23	113	1.65
Under-transcribed		3	0.04	36	0.53	35	0.51	188	2.74	4	0.06	126	1.84
Total additional clusters not within genes		22	0.45	55	1.13	38	0.78	144	2.96	11	0.23	122	2.51
Over-transcribed		18	0.37	36	0.74	21	0.43	53	1.09	9	0.18	51	1.05
Under-transcribed		4	0.08	19	0.39	17	0.35	91	1.87	2	0.04	71	1.46

For each treatment, the number (N) of genes and additional clusters not mapped within predicted genes found significantly differentially transcribed are indicated. For each value, the associated percentage regarding the total number of genes (6850), the total number of clusters not mapped within predicted genes (4868), or the total of genes and additional clusters (11718) is indicated. Genes or clusters were considered significantly differentially transcribed comparatively to controls if their associated P value (Fisher's test) was < 0.001 after multiple testing corrections. Copper: exposed to copper sulfate; Fluo: exposed to fluoranthene; Atraz: exposed to atrazine; Propo: exposed to propoxur; Perm: exposed to permethrin; Imida: exposed to imidacloprid.

### Genes differentially transcribed across treatments

**Figure 2:**
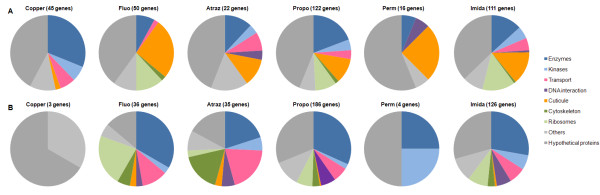
**Genes differentially transcribed in mosquito larvae exposed to xenobiotics**. Analysis was performed on 453 genes found significantly differentially transcribed in at least 1 condition (Fisher's test P_value _< 0.001). Genes were assigned to 9 different categories according to their putative function: enzymes (dark blue), kinases (blue), transport (pink), DNA interaction (purple), cuticle (orange), cytoskeleton dark green), ribosomes (green), others (grey) and unknown hypothetical proteins (dark grey). For each condition, numbers of genes found significantly over transcribed (A) and under-transcribed (B) were compared. Copper: exposed to copper sulfate; Fluo: exposed to fluoranthene; Atraz: exposed to atrazine; Propo: exposed to propoxur; Perm: exposed to permethrin; Imida: exposed to imidacloprid.

Functional analysis of the 453 genes differentially transcribed in mosquito larvae exposed to xenobiotics revealed that genes responding to xenobiotics encode proteins with diverse functions, including a large proportion (up to 50%) of proteins of unknown function (Figure 2 and Additional file 1: Suppl Table 1). Among them, 108 genes were affected by both pollutants and insecticides. Several genes affected by xenobiotics encoded enzymes, cuticular proteins and proteins involved in transport or DNA interactions. As previously shown by PCA, the two chemical insecticides propoxur and imidacloprid, and to a lesser extent the polycyclic hydrocarbon fluoranthene, induce similar functional responses. Response induced by copper appeared distinct compared to organic xenobiotics, with a high proportion of enzymes being over transcribed. Conversely, response to organic xenobiotics was characterized by the overproduction of a large proportion of transcripts encoding cuticular proteins. For these compounds, a positive correlation was found between their lipophilicity (Log Kow) and the proportion of transcripts encoding cuticular proteins being significantly over-produced (r = 0.91; P < 0.01; Log Kow from 0.57 for imidacloprid to 6.1 for permethrin,). Genes encoding cytoskeleton and ribosomal proteins were also affected by various xenobiotics with cytoskeleton proteins showing a marked repression in larvae exposed to the herbicide atrazine. Finally, genes encoding proteins involved in transport were also differentially affected by xenobiotics. A negative correlation was found between the lipophilicity (Log Kow) of organic xenobiotics and the number of transcripts involved in transport being over-produced (r = 0.95, P < 0.01).

### Impact of xenobiotics on transcripts encoding enzymes

**Figure 3:**
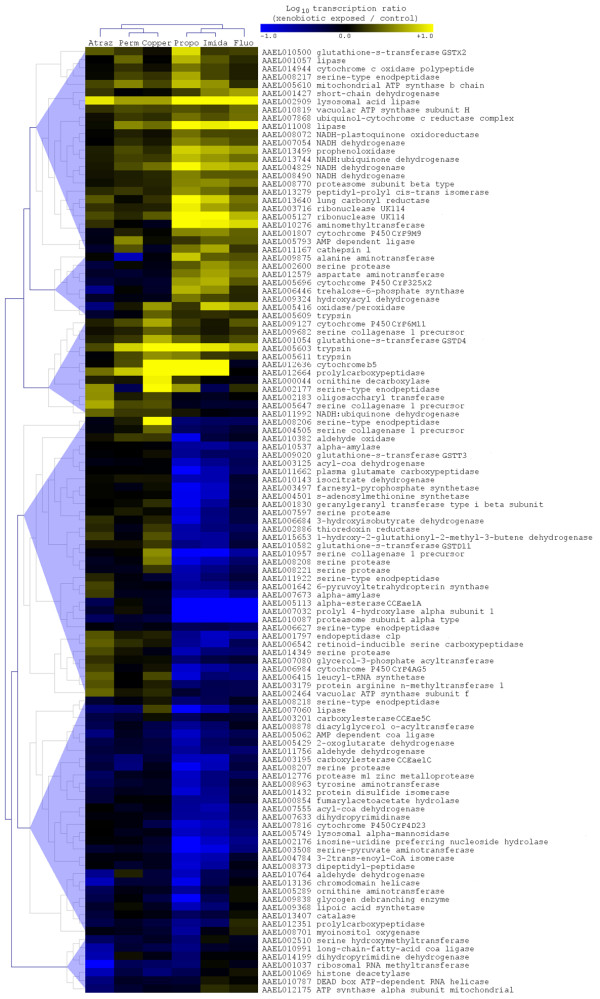
**Enzymes differentially transcribed in mosquito larvae exposed to xenobiotics**. Hierarchical clustering analysis based transcription levels was performed on 115 enzyme-encoding genes showing significant differential transcription (Fisher's test P_value _< 0.001) in larvae exposed to any xenobiotic. Gene tree (left) and condition tree (top) were obtained using Pearson's uncentered distance metric calculated from all Log_10 _transcription ratios (xenobiotic exposed/controls). Color scale from blue to yellow indicates Log_10 _transcription ratios from -1 (10-fold under transcription) to +1 (10-fold over transcription). For each gene, accession number and annotation are indicated. Copper: exposed to copper sulfate; Fluo: exposed to fluoranthene; Atraz: exposed to atrazine; Propo: exposed to propoxur; Perm: exposed to permethrin; Imida: exposed to imidacloprid.

Clustering analysis of genes encoding enzymes significantly differentially transcribed in larvae exposed to xenobiotics revealed that the transcript level of 115 enzymes was affected by one or more xenobiotic (Figure 3). The transcript level of these enzymes was strongly affected in larvae exposed to the insecticides propoxur and imidacloprid and the aromatic hydrocarbon fluoranthene. A gene tree based on transcript levels across all treatments revealed a distribution in 6 main different enzyme clusters mainly influenced by these 3 xenobiotics. Twelve genes encoding enzymes potentially involved in xenobiotic detoxification were found differentially transcribed, including 5 cytochrome P450s monooxygenases (P450s), 4 glutathione S-transferases (GSTs) and 3 carboxy/cholinesterases (CCEs). Among them, the three P450s *CYP9M9 *(AAEL001807), *CYP325X2 *(AAEL005696) and *CYP6M11 *(AAEL009127) were induced by multiple xenobiotics. Interestingly, the *cytochrome b5 *(AAEL012636), a co-factor associated with P450 detoxification systems, was also strongly induced in mosquito larvae exposed to insecticides and copper. Among GSTs, *GSTX2 *(AAEL010500) was strongly and specifically induced by the insecticide propoxur while the induction of *GSTD4 *(AAEL001054) appeared less specific. Transcripts encoding esterases were mostly found under produced following xenobiotic exposure. Finally, several transcripts encoding enzymes involved in the production of energy within the respiratory chain such as NADH dehydrogenase and ATP synthase were over-produced in mosquito larvae exposed to xenobiotics while multiple serine proteases, amylases and peptidases were down-regulated.

## Discussion

### Analyzing transcriptome variations using digital gene expression tag profiling

Following the genome sequencing of the dengue vector *Ae. aegypti*, 15,419 putative genes were identified and transcripts were detected for 12,350 genes by combining cDNA microarray, massive parallel signature sequencing (MPSS) or EST sequencing on several mosquito life stages [21]. By using the DGETP method, we sequenced 29.4 millions 20-mer tags across 7 distinct cDNA libraries obtained from 4^th^-stage larvae. This approach allowed us to detect significant transcription signals for 6,850 predicted genes. Considering that several genes may not be transcribed in 4^th^-stage larvae and that transcripts assayed by the DGETP method require the presence of a DpnII restriction site, such transcriptome coverage appears satisfactory. Besides, sequence variations between the *Ae. aegypti *strain used in our study (Bora-Bora strain) and the one used for genome sequencing (Liverpool strain), led to the rejection of numerous reads. Within our mosquito strain, allelic variations were detected for numerous loci and also led to the rejection of a considerable proportion of reads as only alleles exactly matching to the reference genome sequence were considered in the analysis (see methods). However, we believe that such high mapping stringency is critical for generating accurate gene transcription data with short cDNA tags. Improving the number of reads by replicating sequencing libraries for each sample will allow a better assessment of biological and technical variations together with increasing transcriptome coverage. By sequencing 10 million random 36 bp cDNA fragments from two cDNA libraries of females *Drosophila melanogaster*, Sackton et al. detected 2,540 annotated genes [22]. By targeting a defined region of cDNAs, the DGETP method can generate wider transcriptome coverage together with a higher number of cDNA tags per gene, leading to more precise gene transcription data. Provided a reference genome is available and the aim is to quantify transcript levels between different biological samples, we confirm that methods based on the combination of LongSAGE and next-generation sequencing technologies are perfectly suited for deep transcriptome analysis [15]. Recent improvements in sequencing technologies (~30 million reads/lane on the illumina Genome Analyzer system) are now making sequencing-based approaches the methods of choice for whole transcriptome analyses.

Among the 15,253 20-mer cDNA tags successfully mapped to *Ae. aegypti *genome, 35% were not located within predicted gene boundaries extended by 300 bp at their 3' end (see methods). These tags could be gathered into 4,868 genomic clusters with more than 40% of them showing significant transcription signal (> 100 reads, Additional file 1: Suppl. Figure 1). These clusters may represent genes, exons or UTR extensions not predicted by automated annotation. Recent studies revealed that the genome of complex organisms produce large numbers of regulatory noncoding RNAs (ncRNAs) that can be antisense, intergenic, interleaved or overlapping with protein-coding genes [23,24]. In that concern, it is likely that a significant proportion of transcript signatures detected outside predicted genes represent ncRNAs. The use of next-generation sequencing approaches specifically targeting insect ncRNAs will help decipher their role in mosquito gene regulation and in the capacity of insects to adapt to different environmental conditions.

### Impact of xenobiotics on mosquito larvae transcriptome

Global analysis of transcriptome variations associated with a 48 h exposure of mosquito larvae to low doses of insecticides and pollutants revealed their ability to adjust to modifications of their chemical environment. The number of transcripts affected varies greatly depending on the xenobiotic used for exposure. When considering organic xenobiotics (all but copper), this number increased together with the molarity of the xenobiotics. Our results also revealed that the lipophilicity of the xenobiotics affects the number of differentially transcribed genes encoding cuticular proteins and transporters. It has been demonstrated that lipophilic xenobiotics accumulate in biological membranes or lipid reserves, modifying their distribution across tissues and cells [25,26]. Although our experimental design did not allow segregating between the quantity of xenobiotic and their inherent chemical properties, it is likely that molarity and lipophilicity are key factors affecting the magnitude and the specificity of transcriptome variations observed here.

Our results demonstrated the similar strong transcriptome response of mosquito larvae exposed to the insecticides propoxur and imidacloprid. Despite belonging to two different chemical groups, the carbamate propoxur and the neonicotinoid imidacloprid both potentiate the functioning of nicotinic cholinergic receptors [27]. Although genes encoding the primary targets of these insecticides (acetylcholinesterase or nicotinic receptors) were not found significantly differentially transcribed, the similar transcriptome responses to these two insecticides may be partly related to similar effects generated by the alteration of cholinergic neurons functioning [28,29].

We previously demonstrated that exposing mosquito larvae to various pollutants for few hours can increase their tolerance to insecticides possibly through an induction of detoxification enzymes [11,12,30]. Among the different pollutants tested, polycyclic aromatic hydrocarbons were often the most potent for increasing insecticide tolerance, possibly due to their ability to induce detoxification enzymes [31]. The present study detected a considerable number of genes encoding detoxification enzymes (89 cytochrome P450s, 22 GSTs and 27 carboxylesterases) including several genes showing transcription level variations. However, only a small proportion of them were found significantly affected by xenobiotic exposure, probably due to insufficient number of reads regarding our Fisher's t test P_value _threshold. Among them, members of cytochrome P450 families frequently involved in resistance to insecticides and plant toxins [7-9,32-34] were over transcribed following exposure to fluoranthene, propoxur or imidacloprid. By revealing that several other genes with a broad range of biological functions are similarly affected by insecticides and pollutants, our results suggest that the impact of pollutants on the ability of mosquitoes to better tolerate chemical insecticides might also be the consequence of the induction/repression of other proteins involved in a wide range of functions. In this concern, several cuticular proteins were found over transcribed in mosquito larvae exposed to insecticides or organic xenobiotics. It has been suggested that mosquito may protect themselves from insecticides by cuticular protein thickening leading to a reduction of insecticide penetration [4,35]. Other studies demonstrated that cuticular component deposition is stimulated by environmental stress [36].

Our results also suggest that mosquito larvae exposed to xenobiotics undertake a metabolic stress associated with changes of their chemical environment. Global cellular stress response has been defined as all proteins over-produced due to environmental stress. This response initially named 'general adaptation syndrome' occurs together with increased mobilization of energy from storage tissues [37]. Such stress response has been described for numerous stress factors including exposure to pollutants [38]. In insect cells, response to environmental aggressions can involve various proteins including heat shock proteins [39], metallothioneins [40] or p-glycoprotein synthesis [41]. Although differentiating between xenobiotic-specific and general stress responses is difficult, we also highlighted such protein families including chaperonins, heat shock proteins and ATP-binding cassette transporters (p-glycoprotein family). Moreover, numerous genes encoding enzymes involved in the production of energy or in cellular catabolism such as NADH dehydrogenase, ATP synthase, trypsin and lipases were found over transcribed in mosquito larvae exposed to xenobiotics, confirming a global stress response [37,42].

Significant transcript level variations were observed in response to anthropogenic pollutants though those compounds were not toxic for mosquito larvae (see methods). Although we predicted the relatively important effect of the polycyclic aromatic hydrocarbon (PAH) fluoranthene on mosquito larvae due to known cellular effects on animals [11,12,31,43], responses to atrazine and copper were unanticipated. In animals, the cellular impact of PAHs has been associated with the uncoupling of mitochondrial respiration, direct genotoxic damages and the formation of reactive oxygen species [31,44-46]. The over transcription of NADH dehydrogenase and ATP synthase observed after exposing larvae to fluoranthene confirm that similar effects occur in mosquitoes. Although mosquitoes do not possess the protein targeted by the triazine herbicide atrazine (plastoquinone-binding protein in photosystem II) [47] and a very low concentration was used (10 μg/L), this chemical affected the transcription of several mosquito genes. In plants, atrazine disrupts the electron transport in chloroplasts [48]. In mosquito larvae, several members of the oxidative phosphorylation pathway including NADH dehydrogenase and ATP synthase were induced by atrazine, suggesting a compensation for partial uncoupling of oxidative phosphorylation [44]. Larvae exposed to copper sulfate exhibited a significant over transcription of 45 genes including a large proportion of enzymes while only 3 genes were under-transcribed. The induction of enzymes by copper might be the consequence of chemical interactions between Cu^2+ ^ions and metalloenzymes together with other metalloproteins involved in electron transfers, hydrolysis and oxido-reductions [49-51]. The strong induction of the hemo-protein cytochrome b5 (co-factor of P450s for electron transfer) together with several serine proteases and oxidase/peroxidases support this hypothesis.

## Conclusions

Overall, despite low concentrations, short exposure time and no apparent phenotypic modification, the significant effect of pollutants and insecticides on mosquito larvae transcriptome raise important questions about the 'hidden impact' of anthropogenic pollutants on ecosystems, including mammals. This concern may even be underestimated considering the complex and unknown cross-effects generated by pollutant mixtures often encountered in polluted ecosystems [52]. In nematodes, it has been shown that by applying a realistic heat stress to both uncontaminated and polluted systems, the specimen from polluted environment showed a stronger response [53]. Such effects are likely to occur in polluted mosquito breeding sites and are likely to affect the efficacy of chemical insecticides used for mosquito control [4,5,7,11,12,53]. Although further experiments are required to fully characterize the molecular mechanisms by which pollutants affect insecticide tolerance in mosquitoes, the present study clearly demonstrate that similar response mechanisms are activated by pollutants and insecticides. Finally, the persistent contamination of wetlands by anthropogenic chemicals and the role of phenotypic plasticity in driving selection mechanisms [54] raise the question of the long-term impact of pollutants on the selection of insecticide resistance mechanisms. Additional experiments combining exposure of mosquitoes to pollutants and their subsequent selection with insecticides will provide valuable biological material to answer this question and may later allow improving mosquito control strategies.

## Methods

### Mosquitoes and xenobiotics

A laboratory strain of the dengue vector *Ae. aegypti *(Bora-Bora strain), susceptible to insecticides was reared in standard insectary conditions (26°C, 8 h/16 h light/dark period) and used for all experiments. Larvae were reared in tap water with controlled amount of larval food (ground hay pellets) for 4 days (3^rd ^instar) before exposure for 48 h to 3 chemical insecticides and 3 pollutants belonging to various chemical classes: the pyrethroid insecticide permethrin (Chem Service, USA), the neonicotinoid insecticide imidacloprid (Sigma Aldrich, USA), the carbamate insecticide propoxur (Sigma Aldrich, USA), the herbicide atrazine (Cluzeau, France), the polycyclic aromatic hydrocarbon (PAH) fluoranthene (Aldrich, France) and the heavy metal copper (obtained from CuSO_4_, Prolabo, France). Atrazine is an herbicide heavily used worldwide and is likely to be found in mosquito breeding sites near cultivated areas (e.g. field drainpipes) [30,55]. Similarly, copper is the major component of Bordeaux mixture and is widely used to control fungus on grapes and other berries [56]. Finally, fluoranthene is one of the most ubiquitous PAH and is found at high concentrations in road sediments [57]. Elevated doses of fluoranthene are likely to be found in urban mosquito breeding sites such as road trenches [58] or in oil spillage areas [4].

### Samples preparation

Exposures to all xenobiotics were performed in triplicate with larvae from different egg batches (3 biological replicates per treatment). One hundred larvae were exposed to each xenobiotic in 200 ml tap water containing 50 mg of larval food. Control larvae were obtained simultaneously in similar conditions without xenobiotics. Doses of xenobiotics used for larval exposure were chosen according to the doses likely to be found in highly polluted mosquito breeding sites (INERIS, http://www.ineris.fr). Preliminary experiments revealed that fluoranthene, atrazine or copper did not show any toxicity on mosquito larvae even at higher concentrations than those used in the present study. For insecticides, we chose a concentration resulting in less than 15% larval mortality after 48 h exposure. This low mortality threshold was chosen in order to minimize the effect of the artificial selection of particular genotypes more tolerant to the insecticide during exposure. Doses of xenobiotics used for exposures were 1.5 μg/L permethrin, 40 μg/L imidacloprid, 500 μg/L propoxur, 25 μg/L fluoranthene, 10 μg/L atrazine and 2 mg/L CuSO_4_. After 48 h, larvae were collected, rinsed twice in tap water and immediately used for RNA extractions.

### Preparation of double stranded cDNA tag libraries

For each biological replicate, total RNA was extracted from 30 fresh larvae using the PicoPure™ RNA isolation kit (Arcturus Bioscience, Mountain View, USA) according to manufacturer's instructions. Total RNA quality and quantity were controlled on an Agilent 2100 Bioanalyzer (Agilent, USA). Total RNAs were then diluted to 750 ng/μL in nuclease-free water. For each treatment, total RNAs from the 3 biological replicates were then pooled together in equal proportions. Double-stranded cDNA tag libraries (Additional file 5: Suppl. Figure 4) were prepared by Illumina Corporation. Two μg total RNA were used to isolate mRNAs by using magnetic oligo(dT) beads before cDNA synthesis using superscript II (Invitrogen) at 42°C for 1 h. Second strand cDNAs were then synthesized and mRNAs were removed. Double stranded cDNAs were cleaved at DpnII restriction sites (5'-^↓^GATC-3') and fragments attached to the oligo(dT) beads on their 3' end were purified. Gene expression (GEX) adapters 1 were ligated to the DpnII cleavage sites using T4 DNA ligase (Invitrogen). Double stranded cDNAs containing both GEX adaptors 1 and oligo(dT) beads were then digested with MmeI for 1.5 h at 37°C to generate 20 bp double stranded cDNA tags. These tags were purified before ligating GEX adapters 2 at the MmeI cleavage site using T4 DNA ligase. The adapter-ligated cDNA tag library was then enriched by PCR with two primers annealing to the end of GeX adapters and Phusion DNA polymerase (Finnzymes Oy). PCR cycles were 30 s at 98°C followed by 15 cycles of 10 s at 98°C, 30 s at 60°C, 15 s at 72°C and a final elongation step of 10 min at 72°C. Sequences of primers used for library preparation are available at http://illumina.com. Enriched cDNA tag library was then gel-purified before quality control analysis on an Agilent 2100 Bioanalyzer.

### Sequencing and mapping of cDNA tags to mosquito genome

Each cDNA tag library was sequenced as 20-mers on a genome analyzer I (illumina Corporation). Each cDNA tag library was sequenced on a separated flow cell lane. Sequenced cDNA tags were then filtered from background noise according to their total number of reads across all conditions. Only cDNA tags represented by more than 20 reads were kept for further analysis. Background-filtered cDNA tags were then mapped to the *Ae. aegypti *genome assembly (AaegL 1.1 annotation) using TagMatcher, a software developed in our laboratory and based on the short sequence mapping algorithm 'agrep' [59]. TagMatcher allows matching tags to a reference genome with errors and multiple matching loci (available on request to eric.coissac@inrialpes.fr). After mapping to *Ae. aegypti *genome, only tags without ambiguous nucleotides and mapped without mismatch at a unique genomic location were kept for clustering and differential transcription analysis. To avoid possible bias due to incomplete 3' UTR annotation and because most cDNA tags were expected on the 3' side of genes (see Additional file 5: Suppl. Figure 4), cDNA tags were considered to be 'within' a gene if located between the 5' boundary of a gene and its 3' boundary extended by 300 bp.

### Clustering and differential transcription analysis

In order to collect transcription data from distinct tags matching to a unique transcript or a unique genomic loci without *a priori *knowledge of genome annotation, we clustered tags previously mapped to *Ae. aegypti *genome. Two distinct tags were assigned to a single cluster if *i) *tags were found on the same DNA strand and genomic supercontig, *ii) *tags were separated by less than 500 bp and *iii) *the total number of reads across all conditions was higher for the tag located downstream (3' side) than for the tag located upstream (5' side). The later condition was adopted in order to take in account the effect of partial DpnII digestion of cDNAs during cDNA library preparation, leading to multiple tags located on a single transcript with decreasing number of reads toward the 5' direction (see Additional file 5: Suppl. Figure 4).

Differential analysis of transcription levels in mosquito larvae exposed to each xenobiotic was performed at the gene level for cDNA tags mapped within predicted genes (i.e. gathering all tags mapped within each gene) and at the cluster level for cDNA tags not mapped within predicted genes (i.e. gathering all tags mapped within each cluster). Transcription ratios (TR) were calculated by dividing the number of reads per million (RPM) in xenobiotic-exposed larvae by the number of RPM in control larvae following the formula: TR = [(RPM_treated _+ x)/(RPM_controls _+ x)], where x is a pseudocount equal to 0.2 (approximately 1 read per million per condition). Then, the probability of each gene to be differentially transcribed more than 2-fold in either direction between treated and controls was computed for each condition from raw read counts, taking into account library size. This computation was performed using Fisher's noncentral hypergeometric distribution, which has the advantage over standard hypergeometric law to allow computation of P_value _for a ratio different of one [60]. Holm correction was then applied to multiple test procedure. Genes/clusters were considered differentially transcribed between xenobiotic-exposed larvae and controls if P_value _< 10^-3^.

### Differential effect of xenobiotics on mosquito larvae transcriptome

To compare the global effect of each xenobiotic on *Ae. aegypti *larvae transcriptome, a principal component analysis (PCA) based on Log_10 _TRs was performed on the 453 genes and 225 clusters not mapped within genes showing significant differential transcription following exposure to at least one xenobiotic. Representation of observations (genes and clusters) and conditions (xenobiotics used for exposure) on PCA axis was optimized by applying a Varimax rotation on the 5 axis best representing the variance [61]. A comparative analysis of gene functions differentially transcribed was performed on the 453 genes showing significant differential transcription following exposure to at least one xenobiotic. Genes were classified in 9 different categories: enzymes, kinases, transport, DNA interaction, cuticle, cytoskeleton, ribosomes, others and hypothetical proteins. For each treatment, percentages of genes significantly over- and under-transcribed were compared. To investigate the role of enzymes in the response of mosquito larvae to xenobiotics, a hierarchical clustering analysis based on TRs was performed on the 115 enzymes showing a significant differential transcription. Clustering analysis was performed by loading Log_10 _transcription ratios into TM4 Multi experiment Viewer (MeV) software [62]. Gene and condition trees were calculated using Pearson's uncentered distance metric and complete linkage method with optimization of genes order [63,64].

### Real-time quantitative RT-PCR validation

Transcription profiles of 14 genes were validated by reverse transcription followed by real-time quantitative PCR on same RNA samples used for cDNA library preparation. Four μg total RNAs were treated with DNAse I (Invitrogen) and used for cDNA synthesis with superscript III (Invitrogen) and oligo-dT_20 _primer according to manufacturer's instructions. Resulting cDNAs were diluted 100 times for PCR reactions. Real-time quantitative PCR reactions of 25 μL were performed in triplicate on an iQ5 system (BioRad) using iQ SYBR Green supermix (BioRad), 0.3 μM of each primer and 5 μL of diluted cDNAs according to manufacturer's instructions. Data analysis was performed according to the ΔΔC_T _method taking into account PCR efficiency [65] and using the two genes encoding the ribosomal protein L8 (GenBank accession no. DQ440262) and the ribosomal protein S7 (Genbank accession no. EAT38624.1) for normalisation. For each treatment, results were expressed as mean transcription ratios (± SE) between xenobiotic-exposed larvae and control larvae.

## Data deposition

Detailed transcription data for the 6850 genes detected in the present study are presented in the Additional file 6 (supplementary Table 2).

All next-generation sequencing data and cDNA library informations associated to the present study have been deposited at the EMBL-EBI European Read Archive (ERA) under accession number ERA000115. Experiment metadata are freely accessible at ftp://ftp.era-xml.ebi.ac.uk/meta/xml/ and sequence data are freely accessible at ftp://ftp.era-xml.ebi.ac.uk/vol1/ERA000/ERA000115/. Expression data from the 453 genes found differentially transcribed after xenobiotic exposure are also accessible at http://funcgen.vectorbase.org/ExpressionData/.

All gene accession numbers mentioned in the present manuscript are compatible with Ensembl, NCBI-GenBank and Vectorbase http://aaegypti.vectorbase.org genome databases.

## Authors' contributions

JPD conceived and coordinated the study, participated in sample preparation and data analysis and wrote the manuscript. EC and CM performed bioinformatics and statistical analysis and help to draft the manuscript. RP and MAR performed qRT-PCR experiments, contributed to sample preparation, data analysis and help to draft the manuscript. ACP contributed to data analysis and helped to draft the manuscript. SR contributed to study design, sample preparation, data analysis and helped writing the manuscript. All authors read and approved the final manuscript.

## Supplementary Material

Additional file 1 **Supplementary figure 1**. This figure represents the distribution of the number of reads across distinct genes (6850 genes), clusters not mapped within predicted genes (4868 clusters), all mapped clusters (13118 clusters) and all mapped tags (15253 tags). Genes, clusters and tags are ranked in ascending order according to their total number of reads across all conditions. Click here for file

Additional file 2 **Supplementary table 1**. This table contains all transcription data for the 453 genes found differentially transcribed in *Aedes aegypti *larvae exposed to xenobiotics. Genes are arranged in nine different functional categories: enzymes; kinases; transport; DNA interaction; cuticle; cytoskeleton; ribosomes; others and unknown hypothetical proteins. For each gene, accession number and gene name or annotation are indicated. The number of reads per million (RPM) across all conditions is indicated as an average transcription level. Log_10 _transcription ratios (exposed to xenobiotic/control) are indicated for each xenobiotic relative to control. Transcription ratios with a significant Fisher's test P_value _< 0.001 are shown in bold. Click here for file

Additional file 3 **Supplementary figure 2**. This figure shows the validation of transcription ratios obtained from Digital Gene Expression Tag Profiling (DGETP) by real-time quantitative RT-PCR. Validation was performed on 14 genes found significantly over-transcribed by DGETP in at least one condition. For each gene, transcription ratios from both techniques across all conditions are represented. Black dots represent conditions showing a significant over-transcription in DGETP. Accession numbers and annotations of gene analyzed were: AAEL001626 (zinc/iron transporter); AAEL001981 (serine/threonine kinase); AAEL002110 (cuticular protein); AAEL004748 (pupal cuticular protein); AAEL004829 (NADH dehydrogenase); AAEL005416 (oxidase/peroxidase); AAEL005696 (cytochrome P450 CYP325X2); AAEL005929 (ATP-binding cassette transporter); AAEL010500 (glutathione S-transferase GSTX2); AAEL011008 (lipase); AAEL012636 (cytochrome b5); AAEL013514 (pupale cuticle protein); AAEL009127 (cytochrome P450 CYP6M11); AAEL001807 (cytochrome P450 CYP9M9). Click here for file

Additional file 4 **Supplementary figure 3**. This figure represents the results of the principal component analysis of the effect of xenobiotics on mosquito larvae transcriptome. Analysis was based on log_10 _transcription ratios of all genes and clusters not mapped within genes showing a significant differential transcription in at least one treatment. Both xenobiotic treatments (black dots) and genes or clusters (grey crosses) are represented using the 3 axis best representing the variance. Biplot A: axis 1 and 2 (81.5% of variance). Biplot B: axis 1 and 3 (69.7% of variance). Click here for file

Additional file 5 **Supplementary figure 4**. This figure illustrates the preparation of the double stranded cDNA tag library. Messenger RNAs are isolated by using magnetic oligo(dT) beads before cDNA synthesis. Double stranded cDNAs are synthesized using DNA polymerase I and clived at every DpnII restriction sites. Gene expression (GEX) adapters 1 containing a MmeI recognition site at its 3' side are then ligated to the DpnII clivage sites. Double stranded cDNA fragments containing both GEX adaptor 1 and oligo(dT) beads were then digested with MmeI to generate double stranded cDNA tags. These tags were purified and ligated with GEX adapters 2 at the MmeI cleavage site before enrichment by PCR. Click here for file

Additional file 6 **Supplementary table 2**. This table describes transcription data for the 6850 genes detected in the study. For each gene, accession number and annotation are indicated. For each gene and each condition, reads counts, reads per millions (RPM), transcription ratios (Log_10 _TR) and Fisher's test P_values _are indicated. Click here for file
