# Supplementary figures and images for: Transcriptome response to pollutants and insecticides in the dengue vector Aedes aegypti using next-generation sequencing technology
Source: BMC Genomics. 2010 Mar 31;11:216. doi: 10.1186/1471-2164-11-216 (PMC2867825; doi:10.1186/1471-2164-11-216)

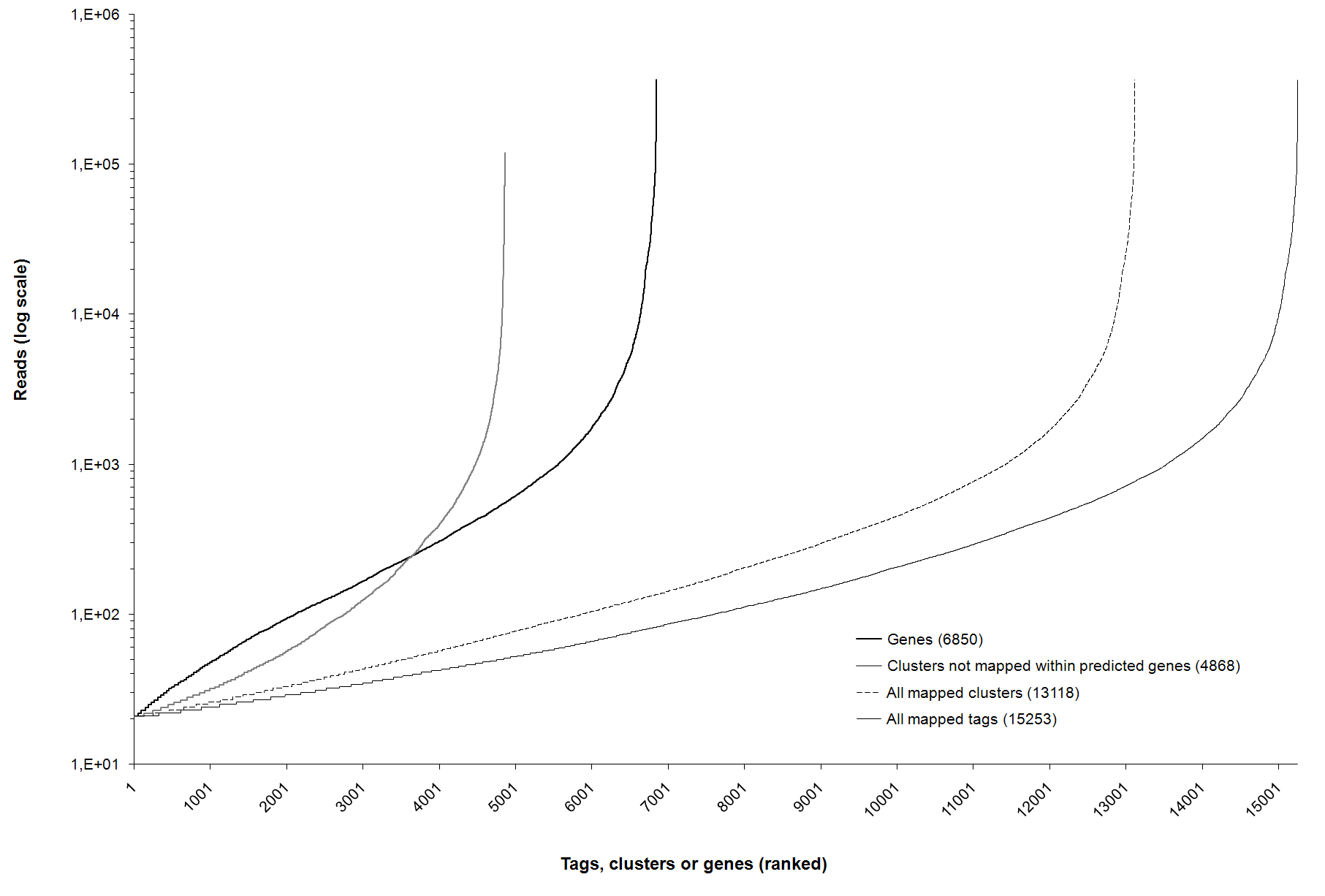

Supplement: Additional file 1 — Supplementary figure 1. This figure represents the distribution of the number of reads across distinct genes (6850 genes), clusters not mapped within predicted genes (4868 clusters), all mapped clusters (13118 clusters) and all mapped tags (15253 tags). Genes, clusters and tags are ranked in ascending order according to their total number of reads across all conditions. [file 1471-2164-11-216-S1.TIFF]

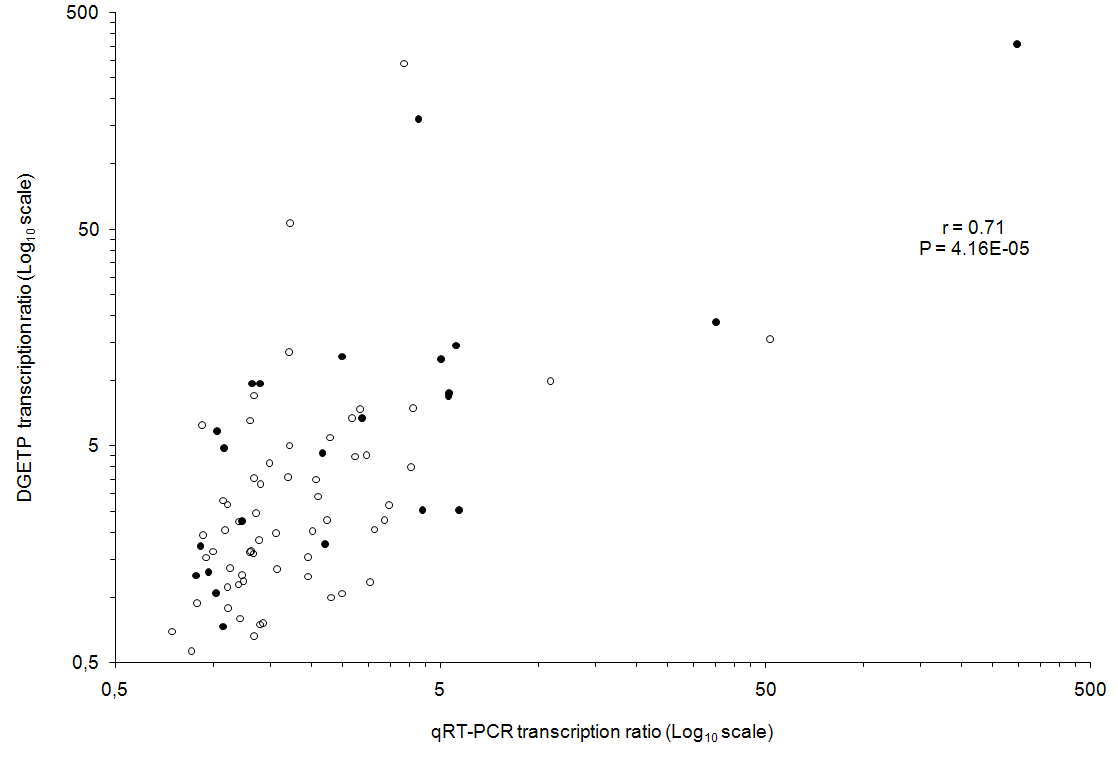

Supplement: Additional file 3 — Supplementary figure 2. This figure shows the validation of transcription ratios obtained from Digital Gene Expression Tag Profiling (DGETP) by real-time quantitative RT-PCR. Validation was performed on 14 genes found significantly over-transcribed by DGETP in at least one condition. For each gene, transcription ratios from both techniques across all conditions are represented. Black dots represent conditions showing a significant over-transcription in DGETP. Accession numbers and annotations of gene analyzed were: AAEL001626 (zinc/iron transporter); AAEL001981 (serine/threonine kinase); AAEL002110 (cuticular protein); AAEL004748 (pupal cuticular protein); AAEL004829 (NADH dehydrogenase); AAEL005416 (oxidase/peroxidase); AAEL005696 (cytochrome P450 CYP325X2); AAEL005929 (ATP-binding cassette transporter); AAEL010500 (glutathione S-transferase GSTX2); AAEL011008 (lipase); AAEL012636 (cytochrome b5); AAEL013514 (pupale cuticle protein); AAEL009127 (cytochrome P450 CYP6M11); AAEL001807 (cytochrome P450 CYP9M9). [file 1471-2164-11-216-S3.TIFF]

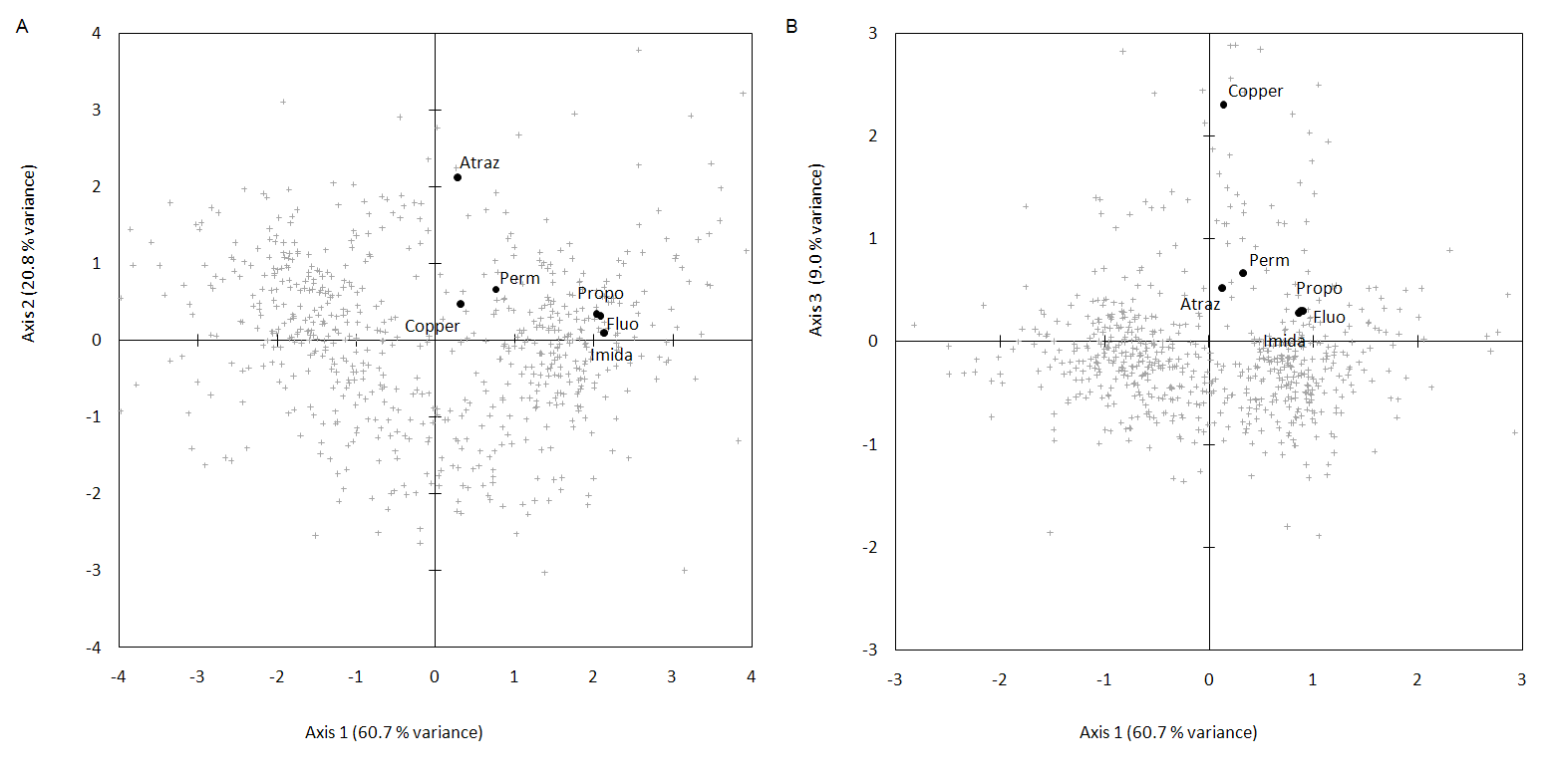

Supplement: Additional file 4 — Supplementary figure 3. This figure represents the results of the principal component analysis of the effect of xenobiotics on mosquito larvae transcriptome. Analysis was based on log10 transcription ratios of all genes and clusters not mapped within genes showing a significant differential transcription in at least one treatment. Both xenobiotic treatments (black dots) and genes or clusters (grey crosses) are represented using the 3 axis best representing the variance. Biplot A: axis 1 and 2 (81.5% of variance). Biplot B: axis 1 and 3 (69.7% of variance). [file 1471-2164-11-216-S4.TIFF]

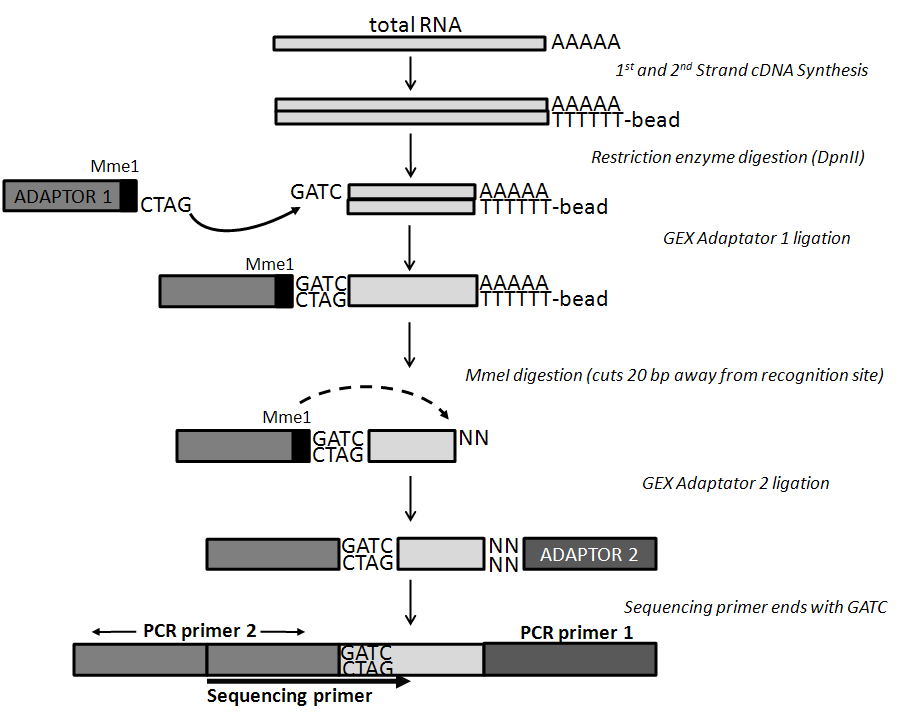

Supplement: Additional file 5 — Supplementary figure 4. This figure illustrates the preparation of the double stranded cDNA tag library. Messenger RNAs are isolated by using magnetic oligo(dT) beads before cDNA synthesis. Double stranded cDNAs are synthesized using DNA polymerase I and clived at every DpnII restriction sites. Gene expression (GEX) adapters 1 containing a MmeI recognition site at its 3' side are then ligated to the DpnII clivage sites. Double stranded cDNA fragments containing both GEX adaptor 1 and oligo(dT) beads were then digested with MmeI to generate double stranded cDNA tags. These tags were purified and ligated with GEX adapters 2 at the MmeI cleavage site before enrichment by PCR. [file 1471-2164-11-216-S5.TIFF]
